# Role of nucleotide‐binding oligomerization domain 1 (NOD1) in pericyte‐mediated vascular inflammation

**DOI:** 10.1111/jcmm.12804

**Published:** 2016-02-24

**Authors:** Rocío Navarro, Pablo Delgado‐Wicke, Natalia Nuñez‐Prado, Marta Compte, Ana Blanco‐Toribio, Gabriel Nuñez, Luis Álvarez‐Vallina, Laura Sanz

**Affiliations:** ^1^ Molecular Immunology UnitHospital Universitario Puerta de Hierro MajadahondaMajadahondaMadridSpain; ^2^ Department of Pathology and Comprehensive Cancer CenterUniversity of MichiganAnn ArborMIUSA; ^3^ Immunotherapy and Cell Engineering LaboratoryDepartment of EngineeringAarhus UniversityAarhusDenmark

**Keywords:** pericyte, NOD1, peptidoglycan, TLR4, lipopolysaccharide, vascular biology, inflammation

## Abstract

We have recently described the response of human brain pericytes to lipopolysaccharide (LPS) through toll‐like receptor 4 (TLR4). However, Gram‐negative pathogen‐associated molecular patterns include not only LPS but also peptidoglycan (PGN). Given that the presence of co‐purified PGN in the LPS preparation previously used could not be ruled out, we decided to analyse the expression of the intracellular PGN receptors NOD1 and NOD2 in HBP and compare the responses to their cognate agonists and ultrapure LPS.

Our findings show for the first time that *NOD1* is expressed in pericytes, whereas *NOD2* expression is barely detectable. The NOD1 agonist C12‐iE‐DAP induced *IL6* and *IL8* gene expression by pericytes as well as release of cytokines into culture supernatant. Moreover, we demonstrated the synergistic effects of NOD1 and TLR4 agonists on the induction of *IL8*. Using NOD1 silencing in HBP, we showed a requirement for C12‐iE‐DAP‐dependent signalling. Finally, we could discriminate NOD1 and TLR4 pathways in pericytes by pharmacological targeting of RIPK2, a kinase involved in NOD1 but not in TLR4 signalling cascade. p38 MAPK and NF‐κB appear to be downstream mediators in the NOD1 pathway.

In summary, these results indicate that pericytes can sense Gram‐negative bacterial products by both NOD1 and TLR4 receptors, acting through distinct pathways. This provides new insight about how brain pericytes participate in the inflammatory response and may have implications for disease management.

## Introduction

Pericytes are key components of the microvascular vessel wall and they are essential for the integrity of the blood–brain barrier (BBB) 1. In contrast to the numerous studies assessing the crosstalk among brain endothelial cells, astrocytes and microglia during inflammation, little is known about the role played by the brain microvascular pericyte. In fact, the response of brain pericytes to pro‐inflammatory stimuli has only been addressed in the last few years 2, 3, 4. In a pioneering work, Kovac *et al*. 2 reported the release of nitric oxide and several cytokines by mouse brain pericytes in response to lipopolysaccharide (LPS), the ligand for the pattern recognition receptor (PRR) toll‐like receptor 4 (TLR4). More recently, we have documented the expression of TLR4 by human brain pericytes (HBP) and characterized the transcriptional profile of LPS‐treated HBP, as well as the signalling cascade beyond TLR4 activation 5. However, Gram‐negative bacteria also contain peptidoglycans (PGN) that are sensed by the PRR nucleotide‐binding oligomerization domain (NOD) 1 and 2 6, 7. NOD1 is expressed by cell types of both haematopoietic and non‐haematopoietic origin, including endothelial cells, where it has been shown to be critical in sensing pathogens 8 and mediating vascular inflammation 9. On the other hand, NOD2 is mostly observed in cells of myeloid and lymphoid origin 7.

It has long been known that highly purified bacterial cell wall components are difficult to obtain using conventional isolation procedures. This implies that results of previous studies, in which standard LPS preparations could contain co‐purified PGN, might be at least partially because of simultaneous activation of TLR4 and NOD signalling. However, expression of the PGN receptors NOD1 and NOD2 in HBP had not been assessed.

To solve these ambiguities, we pursued the following objectives: a) to study the expression of NOD1 and NOD2 in primary HBP; b) to investigate the responses to endotoxin‐free NOD agonists 10 alone or in synergy with ultrapure, PGN‐free LPS; and c) to discriminate TLR4 and NOD signalling pathways in HBP.

## Experimental procedures

### Cell culture and reagents

Primary HBP (#1200) were purchased from ScienCell Research Laboratories (Carlsbad, CA, USA), cultured in pericyte medium (PM) (ScienCell Research Laboratories) and used between passages 3 and 5. HBP are positive for α‐SMA and express the cell surface markers NG2, PDGFR‐beta, CD13, CD73 and CD105 but lack CD31, CD34 and CD45 5, 11. Jurkat clone E6‐1 (TIB‐152) and HL‐60 (CCL‐240) cells were purchased from the American Type Culture Collection (Rockville, MD, USA). TNFα and IFNγ were obtained from PeproTech (Rocky Hill, NJ, USA). LPS‐EK Ultrapure from E. coli K12 and C12‐iE‐DAP were purchased from InvivoGen (San Diego, CA, USA). RIPK2/Src kinase inhibitor PP2 was obtained from Abcam (Cambridge, UK). NF‐κB inhibitor SC514 and MAP kinase inhibitors SP600125 (JNK1‐2 inhibitor), SB203580 (p38 inhibitor) and PD98059 (MEK‐1 inhibitor) were all purchased from Santa Cruz Biotechnology (Heidelberg, Germany). Mouse anti‐NOD1 monoclonal antibody (clone 626919) was from R&D Systems (Minneapolis, MN, USA). Rabbit anti‐NOD1 polyclonal antibodies orb29777 and sc‐99163 were from Biorbyt (Cambridge, UK) and Santa Cruz Biotechnology respectively.

### RNA extraction and cDNA synthesis

HBP were treated with TNFα (50 ng/ml) or with IFNγ (100 ng/ml) for 20 hrs to assess *NOD1* and *NOD2* gene modulation. To study HBP activation, cells were cultured in the presence of C12‐iE‐DAP (5 μg/ml) or LPS (100 ng/ml) for 6 hrs. In synergy experiments, HBP were treated simultaneously with C12‐iE‐DAP (1 μg/ml) and LPS (5 ng/ml) for 6 hrs. To analyse the effect of PP2 on HBP, cells were incubated with the inhibitor (0.01–10 μΜ) for 30 min. before adding C12‐iE‐DAP (1 μg/ml) or LPS (50 ng/ml) for 6 hrs. Total RNA was isolated using the RNeasy Micro kit (Qiagen, Hilden, Germany) and cDNA was obtained using NZY First‐Strand cDNA Synthesis Kit (Nzytech, Lisboa, Portugal).

### Quantitative real‐time PCR (qRT‐PCR)

The following primers were used: human NOD1 forward 5′‐ AAGCGAAGAGCTGACCAAATAC ‐3′ and reverse 5′‐ TCCCAGTTTAAGATGCGTGAG‐3′ and human NOD2 forward 5′‐ ATCGAGCTGTACCTGAGGAAG ‐3′ and reverse 5′‐ GACACCATCCATGAGAAGACAG ‐3′. Primers for IL6, IL8 and SDHA were as previously described 5. All primer sequences were synthesized by Roche Diagnostics (Sant Cugat del Vallés, Spain). qRT‐PCR was performed with a LightCycler 480 apparatus (Roche Diagnostics) using the LightCycler 480 SYBR Green I Master kit (Roche Diagnostics). The level of target gene expression was normalized against SDHA expression. Relative expression of each mRNA was calculated by the ΔCT method as previously reported 5.

### NOD1 flow cytometry

HBP and Jurkat cells were intracellularly stained using the IntraCell kit (Immunostep, Salamanca, Spain) according to the manufacturer's instructions. Cells were incubated with the rabbit anti‐NOD1 policlonal antibody at a dilution of 1:40 for 30 min., followed by 1:100 donkey anti‐rabbit PE‐conjugated secondary antibody (#ab7007, Abcam) and analysed using an EPICS XL flow cytometer (Coulter Electronics, Hialeah, FL, USA).

### NOD1 immunocytofluorescent staining

Cells seeded onto cell chamber slides (Nunc, Roskilde, Denmark) were fixed with 4% paraformaldehyde, permeabilized in 0.1% Triton X100 for 10 min. and incubated with the mouse anti‐NOD1 monoclonal antibody (10 μg/ml) for 1 hr. Then, HBP were incubated with 1:500 goat antimouse secondary antibody, Alexa Fluor^®^ 488 conjugated (#a1101, Invitrogen, Thermo Fisher Scientific, Waltham, MA, USA) for 1 hr. Finally, nuclei were stained with TO‐PRO (Invitrogen). Fluorescence images were captured with a confocal laser scanning microscope TCS SP5 (Leica Microsystems, Mannheim, Germany).

### ELISA

IL8 release was measured in supernatants from the same cells used for RNA isolation with the Human CXCL8/IL8 DuoSet kit (R&D Systems) according to the manufacturer's instructions.

### NOD1 silencing

For NOD1 knock‐down, cells were infected with control copGFP lentiviral particles (Santa Cruz, #sc‐108084), control shRNA lentiviral particles (Santa Cruz, #sc‐108080) encoding a scrambled shRNA sequence or NOD1 shRNA(h) lentiviral particles (Santa Cruz, #sc‐37279‐V) containing three different constructs each encoding target‐specific shRNA. On day 0, HBP were seeded on a 24‐well plate in PM. On day 1, lentiviral particles were added (MOI 7) and incubated overnight. On day 2, cells were selected with 250 ng/ml puromycin (Santa Cruz, sc‐108071). Infection efficiency was monitored by GFP fluorescence and was nearly 90%. At day 4, cells were used for C12‐iE‐DAP (1 μg/ml) stimulation experiments.

### NOD1 Western blotting

Cells were lysed in Laemmli‐lysis buffer (Bio‐Rad, CA, USA) for 10 min. on ice and collected by scraping. Equal amounts of proteins were resolved on 8% SDS‐PAGE gel and transferred onto nitrocellulose membrane using iBlot Dry Blotting System (Invitrogen Life Technologies). Membranes were incubated ON with 0.8 μg/ml of anti‐human NOD1 (sc‐99163) rabbit polyclonal antibody, followed by IRDye800‐conjugated donkey anti‐rabbit antibody, diluted 1:5000 (Rockland Immunochemicals, PA, USA). Simultaneously, anti α‐tubulin mouse monoclonal antibody (T9026, Sigma Aldrich, St. Louis, MO, USA) was added, diluted 1:2000, as a loading control, followed by IRDye700‐conjugated donkey antimouse antibody diluted 1:5000 (Rockland Immunochemicals). Visualization and quantitative analysis of protein bands were carried out with the Odyssey Infrared Imaging System (LI‐COR Biosciences, Lincoln, NE, USA).

### Statistical analysis

Results were expressed as mean ± standard deviation (S.D.). The data were evaluated using the Student's *t*‐test and in some cases by two‐way anova with Bonferroni correction. Values of *P* < 0.05 were considered significant.

## Results

### Expression of NOD1 and NOD2 in human brain pericytes

**Figure 1:**
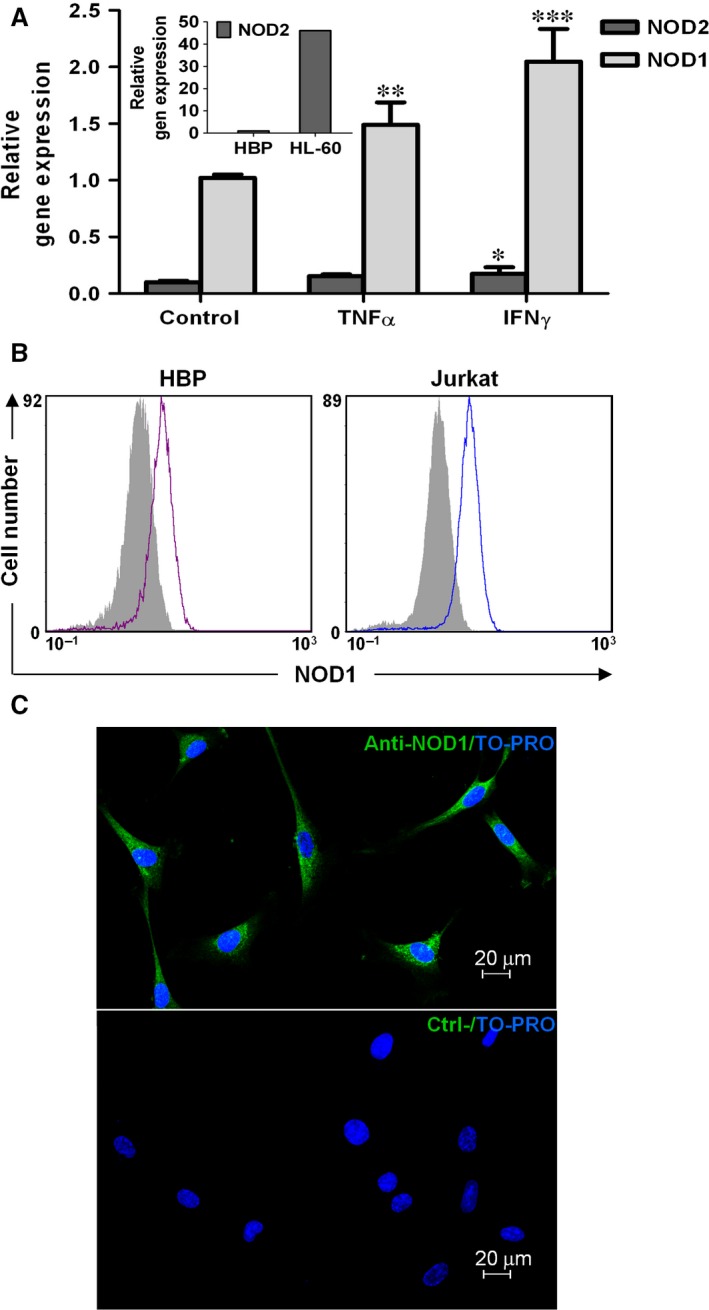
*NOD1* and *NOD2* expression by human brain pericytes. (**A**) Primary HBP were stimulated with TNFα (50 ng/ml) or IFNγ (100 ng/ml) for 20 hrs, and *NOD1* and *NOD2* gene expression was analysed using qRT‐PCR. Data shown are means ± standard deviation (S.D.) of triplicates of two independent experiments (*P < 0.05, **P < 0.01, ***P < 0.001). HL60 cells were used as a *NOD2* positive control (insert). (**B**) FACS analysis of intracellular NOD1 expression in unstimulated HBP and Jurkat cells (positive control). Solid grey curves show negative controls in which the primary antibody was omitted. (**C**) Immunocytofluorescence staining of NOD1, detected in the cytoplasm of unstimulated HBP (top), compared to a negative control in which the same cells are incubated without primary antibody (bottom).

To further characterize the PRR repertoire in HBP, first we analysed gene expression of *NOD1* and *NOD2* by qRT‐PCR (Fig. 1A). *NOD1* was clearly detectable, but *NOD2* was expressed at comparatively lower levels. HL60 cells were used as a positive control of *NOD2* (Fig. 1A, inset).

It has been reported that *NOD1* and *NOD2* expression could be induced by different stimuli, including TNFα and IFNγ, in cell types such as intestinal epithelial cells and keratinocytes 12. After stimulation with 50 ng/ml TNFα, *NOD1* was significantly increased in HBP (*P* < 0.01), but up‐regulation was more evident with 100 ng/ml IFNγ (*P* < 0.001) (Fig. 1A).

Intracellular FACS analysis revealed that NOD1 was expressed at protein level (Fig. 1B). Furthermore, the subcellular distribution of NOD1 was determined after immunocytofluorescence staining under the confocal microscope. NOD1 was constitutively expressed intracellularly, but not on the cell surface, showing a diffuse cytoplasmic pattern (Fig. 1C).

### NOD1 and TLR4 synergize to elicit a pro‐inflammatory response

Next, we addressed whether NOD1 signalling pathway was functional in HBP. To investigate the effect of its agonist C12‐iE‐DAP on the expression of pro‐inflammatory mediators, total RNA from unstimulated or C12‐iE‐DAP‐treated HBP was analysed for *IL6* and *IL8* expression by qRT‐PCR. We compared *IL6* and *IL8* gene modulation by C12‐iE‐DAP with that of ultrapure LPS, not contaminated by other bacterial pathogen‐associated molecular patters (PAMPs).

**Figure 2:**
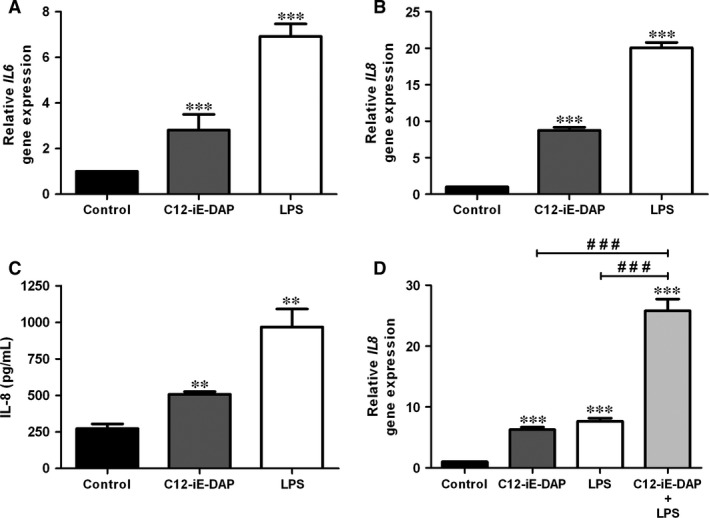
Effect of NOD1 activation in human brain pericytes. HBP were stimulated with C12‐iE‐DAP (5 μg/ml) or ultrapure LPS (100 ng/ml) for 6 hrs and *IL6* (**A**) and *IL8* (**B**) gene expression was analysed using qRT‐PCR. (**C**) In parallel, secretion of IL8 was assessed in cell culture supernatants by ELISA. (**D**) C12‐iE‐DAP (1 μg/ml) and LPS (5 ng/ml) synergistically enhanced *IL8* gene expression by HBP. Results are expressed as means ± standard deviation (S.D.) of triplicates of three independent experiments. (**P < 0.01, ***P < 0.001 ‐*versus* non‐stimulated control; ^###^ *P* < 0.001 ‐*versus* C12‐iE‐DAP or LPS alone).

Both C12‐iE‐DAP and ultrapure LPS induced significant increases in *IL6* (Fig. 2A) and *IL8* (Fig. 2B) gene expression. The up‐regulation with both agonists was more evident for *IL8* than for *IL6*, as previously reported in HBP treated with standard LPS preparations 5. Then, we studied IL8 protein production in the supernatant of the same HBP used for RNA isolation. As shown in Figure 2C, HBP exhibit basal secretion of IL8 without stimuli as assessed by ELISA. Consistent with the RNA results, both C12‐iE‐DAP and ultrapure LPS induced a statistically significant increase in IL‐8 release (Fig. 2C). As emerging evidence suggests cooperative effects of PRR, we also addressed the outcome of combined triggering of NOD1 and TLR4 in HBP, using low concentrations of their respective agonists. When cells were stimulated with 1 μg/ml C12‐iE‐DAP or 5 ng/ml LPS, there was a moderate (although statistically significant) increase in the levels of *IL8* mRNA. However, when HBP were costimulated with C12‐iE‐DAP and LPS at the same concentrations, there was a marked synergistic increase in *IL8* gene expression (Fig. 2D). Interestingly, simultaneous treatment with 5 ng/ml LPS and C12‐iE‐DAP permitted the induction of higher amounts of IL8 RNA than those obtained with a high dose (100 ng/ml) of LPS alone (Fig. 2B).

### Effect of NOD1 silencing

**Figure 3:**
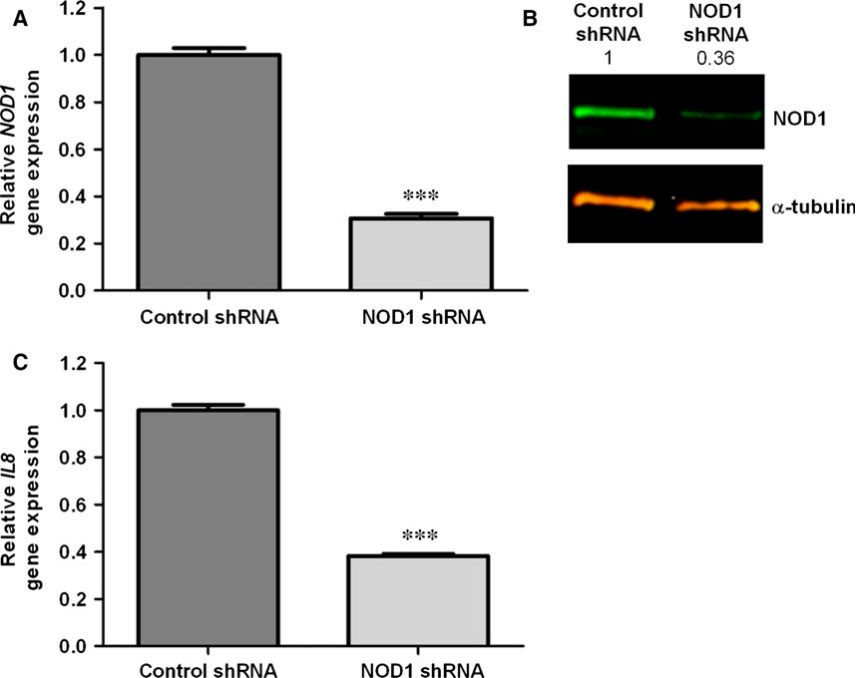
Knock‐down of NOD1 abrogates C12‐iE‐DAP response. (**A**) The knock‐down efficiency of HBP transduced with NOD1 shRNA or control shRNA lentiviral particles was assessed by qRT‐PCR. (**B**) Western blot analysis was used to verify knock‐down of NOD1. Alpha‐tubulin was used as a loading control. Numbers indicate the expression fold change relative to the loading control. (**C**) NOD1 and control shRNA expressing HBP were treated with 1 μg/ml C12‐iE‐DAP for 6 hrs. Data are presented as fold change of *IL8* expression compared to non‐stimulated cells. Data represent mean ± standard deviation (S.D.) of three independent experiments, each performed in triplicates. (***P < 0.001).

To further assess the role of NOD1 in C12‐iE‐DAP‐mediated gene induction, we used shRNA to knock‐down NOD1 expression. Expression of the NOD1 shRNA in HBP resulted in a distinct decrease (>69%, *P* < 0.001) of *NOD1* gene expression as assessed by qRT‐PCR (Fig. 3A). The down‐regulation of NOD1 mRNA was paralleled with a decrease in NOD1 protein content in Western blot (Fig. 3B). Densitometric analysis of protein bands showed a knock‐down efficiency >63%. Moreover, treatment of these cells with C12‐iE‐DAP revealed a statistically significant reduction (>62%, *P* < 0.001) of *IL8* induction (Fig. 3C). These data confirm that C12‐iE‐DAP‐mediated *IL8* up‐regulation in HBP requires functional NOD1.

### **Effect of the RIPK2 inhibitor PP2 on NOD1‐ and TLR4‐mediated responses**

**Figure 4:**
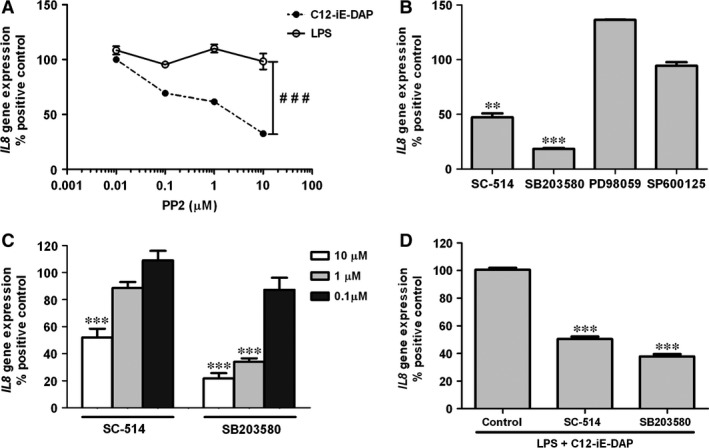
Role of RIPK2, NF‐kB and MAP kinases on NOD1‐mediated responses. (**A**) HBP were incubated with different concentrations of PP2 ranging from 0.01 μM to 10 μM for 30 min. prior to treatment with LPS (50 ng/ml) or with C12‐iE‐DAP (1 μg/ml) and analysed for *IL8* gene expression. Results are expressed as a percentage of response to the agonist alone *versus* non‐stimulated controls (^###^ *P* < 0.001). (**B**) Effect of pre‐treatment with the inhibitors SB203580 (p38 inhibitor), SC‐514 (NF‐κB inhibitor), SPS00125 (JNK inhibitor) and PD98059 (MEK‐1 inhibitor) at 10 μM on response to C12‐iE‐DAP (1 μg/ml). (**C**) Dose‐dependent effect of SB203580 and SC‐514 (0.1–10 μM) on HBP activation by C12‐iE‐DAP. (**D**) Synergistic effects of C12‐iE‐DAP+LPS were inhibited by SB203580 and SC‐514. HBP were pre‐treated with 10 μM SB203580 or SC‐514 prior to stimulation with C12‐iE‐DAP (1 μg/ml) and LPS (5 ng/ml). Data are expressed as mean ± standard deviation (S.D.) and represent three independent experiments, each performed in triplicates (**P < 0.01, ***P < 0.001).

NOD1 receptor signalling occurs *via* receptor‐interacting RIPK2 13, but the functionality of this pathway in HBP had not been previously studied. To further discriminate NOD1 and TLR4 responses in HBP, we used PP2, a potent inhibitor of RIPK2 14. HBP were pre‐treated for 30 min. with increasing concentrations of PP2, ranging from 0.01 μM to 10 μM, and then were stimulated with C12‐iE‐DAP or ultrapure LPS. Treatment of HBP with PP2 resulted in a concentration‐dependent inhibition of C12‐iE‐DAP‐induced *IL8* gene up‐regulation, as assessed by qRT‐PCR. However, inhibition of RIPK2 had no effect on LPS‐induced *IL8* modulation when compared to the non‐PP2‐treated controls, demonstrating a selective inhibition of the NOD1 signalling cascade (Fig. 4A).

### NOD1 downstream mediators in C12‐iE‐DAP‐mediated responses

In additional studies to evaluate which of previously established downstream signalling components of NOD1 pathway were relevant to *IL8* up‐regulation, HBP were treated with 10 μM SC‐514 (NF‐κB inhibitor), 10 μM SB203580 (p38 inhibitor), 10 μM SP600125 (JNK1‐2 inhibitor) or 10 μM PD98059 (MEK‐1 inhibitor) for 30 min. and then stimulated with C12‐iE‐DAP (1 μg/ml). As shown in Fig. 4B, pre‐incubation of HBP with SP600125 or PD98059 did not impair *IL8* expression. However, addition of the p38 or NF‐κB inhibitors to cultures resulted in statistically significant inhibition of C12‐iE‐DAP‐induced *IL8* up‐regulation (*P* < 0.001 and *P* < 0.01 respectively). This inhibition was dose dependant as assessed using a 0.1–10 μM range of each compound (Fig. 4C). Increase in *IL8* mRNA levels was only significantly inhibited at the highest concentration of SC‐514. On the contrary, SB203580 had a potent effect at lower concentrations (*P* < 0.001 at 1 μM). When 10 μM SC‐514 or 10 μM SB203580 were added prior to simultaneous activation with LPS and C12‐iE‐DAP, the synergistic effect on *IL8* up‐regulation was abolished in similar percentages (Fig. 4D).

## Discussion

HBP occupy a strategic position at the interface between blood and the brain to initiate inflammatory processes as a response to systemic infection or blood‐borne PAMPs. Indeed, HBP seem to be well equipped to do so with a growing array of PRR. We have previously reported the expression of TLR4 by HBP; here, we show for the first time that the intracellular receptor NOD1 is functionally expressed in HBP and it is required for recognition of the PGN moiety C12‐iE‐DAP.

It has been long known that the mechanism of NOD1 activation in monocytes and macrophages involves RIPK2. Taking advantage of the fact that the TLR4 pathway is not dependent on RIPK2 15, we were able to discriminate TLR4 and NOD1 signalling in HBP by pharmacological targeting of RIPK2 using the specific inhibitor PP2. Therefore, we have demonstrated that both pathways are functional in HBP, and could rule out that the effect observed with a standard LPS preparation in our previous work was because of co‐purified PGN. Interestingly, TLR4 and NOD1 downstream mediators also offer a distinct pattern. In the HBP response to C12‐iE‐DAP, p38 and to a lesser extent NF‐κB seem to be involved. On the other hand, we have recently described that NF‐κB signalling pathway, but not p38, is activated by LPS in HBP 5. It has been reported that NOD1 and NOD2 agonists significantly enhanced *in vitro* TLR‐induced cytokine secretion by monocytes and dendritic cells 16. Although the effect of C12‐iE‐DAP in HBP was comparatively weak in relation with that of ultrapure LPS, a synergistic effect was observed upon simultaneous treatment with C12‐iE‐DAP and suboptimal concentrations of LPS that induced more *IL8* up‐regulation than high‐dose LPS alone. However, the molecular mechanisms responsible for this crosstalk between PRRs are not well known. Pre‐treatment with p38 or NF‐κB inhibitors of HBP simultaneously stimulated with LPS and C12‐iE‐DAP confirmed the involvement of both pathways in the synergistic effect.

Recent reports have documented the activation of pericytes by pro‐inflammatory factors released by ‘professional’ immune cells, such as IFNγ, TNFα and IL1β 3, 4. Indeed, *NOD1* expression is up‐regulated by IFNγ and TNFα in HBP. Beyond this passive role in the amplification of inflammatory responses, expression of functional TLR4 and NOD1 suggests that brain pericytes are endowed with the capacity of sensing PAMPs, directly contributing to the onset of innate immune responses. Release of cytokines and chemokines by pericytes may trigger paracrine and autocrine signalling pathways contributing to BBB disruption, being this a feature not only of brain inflammation but also of early neurodegenerative disorders. Drugs modulating the pro‐inflammatory activity of pericytes may provide a novel strategy to restore BBB integrity and reduce brain injury in a variety of pathological conditions.

## Conflict of interest

The authors confirm that there are no conflicts of interest.
